# The Institutional Worker

**Published:** 1913-04-12

**Authors:** 


THE
### INSTITUTIONAL WORKER
**Being a Special Supplement to "The Hospital"**

### Married Officers.

You are perfectly justified in protesting against the common form of advertisement for a vacant post stipulating there shall be no "encumbrances." There is need for a general levelling up in institutions throughout the country of quarters for married officers. There is no doubt these quarters should be situated outside the main building, but within a short distance in the grounds, and that with a trifling expenditure much could be done to remove the present galling disabilities. No class of officer is more valuable than the young married man with a growing family, and it is very disastrous that he should be deterred from remaining in the service.—Disgusted.

### The Defective Colony.

Classification is the secret of success in dealing with the feeble-minded, and although there is something to be said for your plea for small cottage-homes, we have no hesitation in preferring a combination of various districts in providing accommodation. Unless numbers are large, the cost of adequate classification is prohibited. At Monyhull nearly 400 children are received, and the courses of work and recreation are adapted to every grade of intelligence. If the defectives are herded together and merely provided with food, clothing, shelter, and mechanical employment, the results cannot fail to be deplorable.—V. D.

### Algerian Hospital.

Private patients are received in the British Cottage Hospital at Algiers, in the Nursing Home attached, at a charge of from 15 to 30 francs a day, and nurses can be obtained from here, so that you need be under no apprehension on this score. Medical appliances can also be hired from the hospital, and it is a great source of protection and help to visitors. Five nurses are employed during the season, at a charge of 75 francs a week to non-subscribers and 50 francs to subscribers. The management is thoroughly practical and good.—Viator.

### Nursing Agency in Lucerne.

There is no institute for the supply of English nurses in Lucerne, as the demand is intermittent, and hotel invalids generally bring their own nurses.

### OUR BUREAU OF INFORMATION.

### Rules for Correspondents.

1. Every letter, on whatever subject, must be accompanied by the coupon to be cut from the back cover (inside page) of The Hospital, current issue, and must contain the name and address of the correspondent with pseudonym for publication if desired.
2. Replies by post cannot be given, save under exceptional circumstances at the Editor's discretion.
3. Letters from Approved Homes in reply to special needs published in the Bureau should state terms and full particulars, and be sent prepaid under cover to the Editor of the Bureau with name written across coupon for identification.
4. Proprietors of Homes which have not yet been entered on the List of Approved Homes, but have spare accommodation likely to suit special needs, are invited to write for an application form for registration. The fee for registration, which includes two announcements of the Home in the Bureau and other privileges, is 10s.
5. All communications to be addressed to the Editor of The Hospital, 28 Southampton Street, Strand, London, W.C., and marked "Bureau of Information."

### Invitation to Readers.

**The Editor is prepared to make known without charge the needs of any who may be sick or in difficulties, and to guide them in making choice of Homes for treatment or convalescence. Reduced terms can often be arranged with Homes on the Approved List for those unable to pay full fees. Queries are specially invited from those who are engaged in any kind of philanthropical work. A new edition of the leaflet describing the purposes of the Bureau of Information is now ready and will be sent free of charge on application.**

### Electrical Treatment.

It would be quite beyond the limits of space at our disposal to furnish you with "a list of all the towns in England that have doctors who give electrical treatment." You will find it practicable to get static and high-frequency current in every place where there is a good general hospital, and you can obtain the name of the physician to the electrical department in each case from the Secretary.—Hilda.

### Class Preparation for C.M.B.

As you are already a fully trained nurse, we do not think it will make much difference to your subsequent prospects as a private nurse whether you are trained for the C.M.B. Examination in a maternity hospital or through outside lectures, etc. It depends very much on the particular doctors you work under, but as a rule you would find the C.M.B. certificate accepted without question. The best way to procure a post "in a Nursing Home or as staff nurse in a hospital for six or eight weeks," to fill in time, is to advertise for a holiday engagement. Nurses to fill gaps are much in request from now on through the summer.—Nurse E. M.

### The Cost of a Licence.

Only one patient (certified) can be received in an unlicensed house, except by special consent of the Commissioners in Lunacy, who may allow one more for the benefit of the patient. The fee for a licence is 10s. per private patient, but with a minimum of £15. There is a 10s. stamp,

Nurses, when wanted, command from 8s. to 12s. a day. You could obtain information about prospects by writing to the Red Cross, Lucerne. If you want to spend the summer there, and get work to pay your expenses, we advise you to call and see all the hotel proprietors on arrival, and report yourself at the British Consulate.—S. F. M.

### Nurses in Alexandria.

Write to the Matron, Victoria House, Alexandria, but we fear she will not be able to give you a very encouraging report. Formerly this Home employed two nurses, but this has been now given up. Nurses who are out of work can be received. We understand there is always a sufficient supply of nurses in the place, and that constant employment cannot be guaranteed. There are no private Nursing Homes, nor are they needed. Of course, you know it is far too late now to go out.—Janet.

### A Substitute for Sweetbreads.

These are certainly dear, yet are often ordered for convalescents. Where money has to be specially considered, use, instead of sweetbreads, calves' or sheep's brains. They are light, digestible, nourishing, and few will be aware of the little fraud. Try these recipes:—

### Sheep's Brains on Toast.

Soak one or two sets of brains in cold, salted water for an hour; then remove skin and fibres. Put the brains into cold water, with salt, on the fire and boil for five minutes; then drain off this water, and cook them in stock or milk for about twenty minutes. Cut a piece and see it is free from any pink tinge inside, and, if not, chop them finely, removing any skin. Spread the chopped mixture on hot, buttered toast, dust with seasoning and a little chopped parsley to give a pleasing touch of colour.

### Sheep's Brains with Egg Sauce.

Prepare and cook as for the toast, but do not chop the brains. Instead, lay them on a hot dish and strain over a gill of plain melted butter sauce to which has been added the raw yolk of an egg, and half a teaspoonful each of lemon juice and chopped parsley.—H. M. M., Somerset.

### Laundry at a Women's Hospital.

THIS is a special hospital for women; beds 53; during 1912 average number occupied 48; staff 34. Acute surgical cases, and five maternity cases are received. Major operations sixteen to eighteen a week.

All our washing is done by an outside laundry by contract at 1d. per article.

(*a*) Average cost of patients' washing per week, 1s. 8½d. per head.

(*b*) Staff 6d. per head; this includes all bed and table linen, towels, curtains, and household washing. The nursing staff are allowed 2s. each, and the domestic staff 1s. 6d. each per week for personal washing.—FLOSS.

### Address of German Physician.

IF you will write to Dr. Mehnarto, in our care, we shall be glad to forward the letter to him. We further refer you to a letter published in the *Lancet* of March 8, p. 719, signed by Dr. Mehnarto and four well-known medical men.—T. K. AND F., LTD.

### Homes Wanted.

WE have been asked by the Secretary of a Society for the Employment of Women for the names of inexpensive Nursing Homes where ladies could be taken who are in poor circumstances. We shall be happy to forward letters from any of the Approved Homes on our List who would like to send particulars of their terms. We understand that a great need exists for such places, which "would have no difficulty in obtaining a steady supply of patients." We do not feel so confident about this as our correspondent, but it may be worth while to inquire about it.—M. G. S. (158A).

### Hospital Visiting.

COMMUNICATE with the matron of the hospital or infirmary you desire to visit, and ascertain whether there is a vacancy in any ward for a visitor. Should you find that your help is not immediately required, we advise you to write to the Secretary of the Society for the Visitation of the Sick, 112 St. Martin's Lane, Trafalgar Square, London, who will probably be very glad to utilise your services under the very definite conditions which have been found advisable in this work. There is an opening for much valuable personal service in this connection, but it needs discretion and the strict subordination of private opinions and plans to conform with regulations.—PITIFUL.

so that a private asylum receiving any number of patients from three to thirty pays £15 10s. a year for licence. You know, perhaps, that no new licences are issued, so that if you desire to have a licensed house you must buy or otherwise obtain one already existing. The authorities for licences are the Commissioners in Lunacy in London and the justices in the provinces. Application is made to the latter at Quarter Sessions. You may, of course, take mild cases, nervous patients, etc., who are not certified without a licence.—R. G. C.

### Liability for Board Wages.

A CORRESPONDENT, signing herself "Anxious One," writes: I was abruptly called upon by registered letter to resign "at once." I replied that as I had never heard one word of complaint from the committee, not even a word of warning, I declined to resign or to accept my resignation unless the committee could give me a sufficient reason for same. A special meeting of the committee was held, and the matter was deferred for three months (I agreed to give or take three months' notice when appointed). I am now asked to take three months' money in lieu of notice. Can I claim board wages for the three months? The committee have given me a testimonial saying they found me a capable and economical house manager, energetic and hard-working, and kind to the patients under my care. All the medical staff have given me testimonials to the effect that I am a conscientious and capable nurse. [We have consulted legal opinion, which is that as "Anxious One" agreed to take three months' wages in lieu of notice, this appears to preclude her from claiming anything more. At any rate, it has been decided that a domestic servant who is discharged without reason is entitled to the wages accruing up to the time of her discharge, and to a calendar month's wages in addition, but not to board wages for the month. Of course, if it could be shown that there was wrongful dismissal, the profits lost owing to that dismissal could be recovered. Thus it has been laid down in such cases that the damages are to be measured by the amount of wages which the servant has been prevented from earning by reason of his wrongful dismissal, including the value of any other benefit to which he is entitled by virtue of his contract, and of which he is deprived in consequence of its breach. In view of the provision as to three months' notice, no question of damages can arise in the present case.]

### Institution Facts and Figures.

### April Question.

STATING the number of beds in your institution and the class of patients received, give the following particulars:—

The number of garments washed during any four weeks in the winter months, with the price per unit, and the manner in which the cost is arrived at. In addition to wages, fuel, light and power, interest on capital should be taken into account. State the quantity, description, and cost of soap used in laundry for the same month. If curd and oil soap be used, the amount of alkali should also be stated. State also the quantity of starch and blue.

The best answers will be published, and for each contribution considered by the Editor to be of sufficient interest for publication payment of FIVE SHILLINGS will be made.

All letters to be addressed, with coupon enclosed, to The Editor, The Bureau of Information, 28-29 Southampton Street, Strand, London, W.C., and to be received by the last day of the current month.

### Cost of Building Small Hospital.

TURN to Chapter X. of Burdett's "Cottage Hospitals," published by The Scientific Press, Southampton Street, Strand. There you will find the plans of several model small or cottage hospitals, with the cost of building and equipping each. Other chapters in this book deal with considerations to be kept in view in building and fitting up such a hospital, the sanitary arrangements, the working, nursing, and the question of patients' payments. We think a careful perusal of this book will answer all your points, but if not, we shall be glad to hear from you again.—SUSSEX.

### Recovery from Insanity.

YOU should apply to the After Care Association for Poor Persons Discharged Recovered from Asylums for the Insane, whose address is Church House, Dean's Yard, Westminster. But they do not interest themselves in any case unless it is certified as absolutely recovered. The Association finds suitable work for the applicant, and would, in the case you mention, which seems a most deserving one, supply the necessary clothes to enable the man to take up work. We would advise you not to delay in endeavouring to get suitable employment for your case, as any anxiety which may be suffered as to material wants has a very bad effect on cases of mental disorder.—J. D.

### The Editor's Letter-Box.

### Communications have been received and attended to from:—

Dr. Roberts, N. Chambers, Dr. E., I. Jeffery.

J. C. GREEN.—Thanks for the letters you forward us; we regret you were troubled, and note you would be willing to receive two invalid children at not less than 18s. 6d. each per week.

INVALID CHILDREN'S AID ASSOCIATION. — Very glad to know that you find our Approved List of Homes able to receive children of service.

J. B., Maidstone.—Happy the little advance pension furnished by the friends of the Bureau is adding to your comfort. We have forwarded your second letter to a lady interested in your case. Thanks for calling our attention to the article you refer to.

### Editor's Notices.

**Contributions:**

Contributions should be written, or preferably typed, on one side of the paper only, and all articles sent in are accepted upon the distinct understanding that they are forwarded to The Hospital only.

The Editor cannot undertake to return MSS. not used, but when a stamped directed envelope is enclosed the MSS. may be returned if a special request is made.

Accepted articles and paragraphs of news will be paid for after publication at the scale rate.

**Address:**

To prevent delay all contributions and letters on editorial business must be addressed exclusively to the Editor, "The Hospital," 29 Southampton Street, Strand, London, W.C. It is important that this regulation shall be strictly observed.

**Correspondence:**

Correspondence on all subjects is invited. The name and address of correspondents must be given as a guarantee of good faith, but not necessarily for publication.

**Special Articles:**

Special articles are invited, and questions, inquiries, and paragraphs upon all matters relating to the work, administration, and management of general, special, mental, fever, cottage and convalescent hospitals, sanatoria, homes, institutions, societies and organisations for the treatment or care of the sick, injured and dependants of all classes. Every matter affecting the interests and welfare of the staff of all grades working in these institutions will receive special consideration.

**Administrative Medicine, Research and Items of News:**

Special terms will be paid for approved articles on subjects in this wide field by experts who have made some section of it their special study and interest. We wish to give prominence to every movement tending to advance accuracy of diagnosis, efficiency in treatment, and the development of modern methods to eradicate disease and promote the welfare of the suffering.

**A Bureau of Information:**

We invite inquiries and applications for information and help in providing for that numerous class of sufferers who require special care or house-room which they cannot obtain in their own homes or secure for themselves. We cannot, however, prescribe, or recommend practitioners

**Books for Review:**

Publishers are particularly requested to send advance proofs of any new books of importance whenever possible, as the Editor has made arrangements to publish immediate reviews on a new plan.

**Photographs, Plans, Blocks, and Illustrations:**

It is requested that wherever possible MSS. may be accompanied by illustrations in any of the above forms. The name of the sender and of the article to which it belongs should be written on the back of each photograph, drawing, or block for purposes of identification.

**Local Papers:**

Newspapers containing reports or news paragraphs should be marked and addressed to the Sub-Editor.

**The Coupon System:**

A coupon will be found at the bottom of the third inside page of the cover each week. This coupon must be attached to every question or inquiry to which an answer is desired in The Hospital.

### A Great Help.

In consequence of the ever-increasing circulation of The Hospital it is often a matter of considerable difficulty to obtain a copy of the current issue at the local newsagents. In these circumstances we strongly recommend you to guard against the contingency of The Hospital being sold out by sending a subscription to the Journal either through a bookseller or direct to the Publishing Offices. By this means it is possible to secure a copy of the Journal almost immediately upon publication.

This is *a great help* when seeking a post, as it enables you to send in your application without delay, an advantage which may secure you the appointment. In addition to this, as a subscriber, no matter how many times you may change your address, or in what part of the country you may be at the moment, a postcard will ensure that you receive The Hospital on the same day and at the same hour as when at home.

**Subscriptions** may begin at any time, and are payable in advance. Cheques and Post-Office Orders should be crossed London County and Westminster Bank, Covent Garden Branch, and made payable to the Manager of The Hospital.

Rates of Subscription (payable in advance).

| | | |
|---|---|---|
| **UNITED KINGDOM.** | | |
| Three Months (including Postage) | ... ... ... ... | 2s. 0d. |
| Six Months | do. ... ... ... ... | 4s. 0d. |
| Twelve Months | do. ... ... ... ... | 6s. 6d. |
| **FOREIGN AND COLONIAL.** | | |
| Three Months (including Postage) | ... ... ... ... | 2s. 6d. |
| Six Months | do. ... ... ... ... | 5s. 0d. |
| Twelve Months | do. ... ... ... ... | 8s. 8d. |

*SUBSCRIPTION FORM.*

*Please send me* The Hospital *for..............months, commencing with your issue of.............................., for which please find enclosed.............................*

*Name*......................................................

*Address* ...................................................

.............................................

*Date*.....................................

*To* .................................... *Newsagent.*

### Manager's Notices.

**Letters relating to the publication, sale, and advertising departments of "The Hospital" must be addressed to The Manager, "The Hospital," The Hospital Building, 28 and 29 Southampton Street, London, W.C.**

**Advertisements:**

To ensure insertion, all advertisements for The Hospital must reach the Manager not later than Wednesday morning in each week. For scale of charges see pages v and 4.

**Remittances:**

**It is especially requested that remittances be made by Cheque or Postal Order, and in halfpenny stamps only when the amount is under sixpence.**

YOU MUST HAVE THE

LATEST EDITION

OF

THE NURSE'S PRONOUNCING DICTIONARY

OF

MEDICAL TERMS AND NURSING TREATMENT

Compiled by

HONNOR MORTEN

Seventh Edition.

Revised and Edited by Mary I. Burdett.

BECAUSE:

IT IS QUITE UP TO DATE.

CONTAINS NO OBSOLETE TERMS.

IS ABSOLUTELY DEPENDABLE.

The proofs were passed by a highly Qualified Medical Man, consequently the utmost reliance can be placed in the information within its pages.

GET A COPY TO-DAY.

Of all Booksellers, or direct from THE PUBLISHERS,

THE SCIENTIFIC PRESS, Ltd.,
28 & 29 Southampton Street,
Strand, London, W.C.

